# Disparities in access to endovenous treatment options in chronic lower extremity superficial venous insufficiency: A national 7-year analysis

**DOI:** 10.1016/j.jvsv.2024.101867

**Published:** 2024-03-05

**Authors:** Shin Mei Chan, Azadeh Tabari, Emma Rudié, Brian D'Amore, Meredith Cox, Ayah Mugahid, Shams Iqbal, Dania Daye

**Affiliations:** a UCSF Department of Radiology & Biomedical Imaging, University of California, San Francisco, CA; b Department of Radiology, Massachusetts General Hospital, Harvard Medical School, Boston, MA; c Drexel University College of Medicine, Philadelphia, PA; d Duke University School of Medicine, Durham, NC

**Keywords:** Superficial venous disease, Chronic venous disease, Ethnicity, Race, Age, Endovascular thermal ablation, Venous stripping, Radiofrequency ablation

## Abstract

**Objective:**

The goal of this study was to analyze trends in treatment access for chronic superficial venous disease and to identify disparities in care.

**Methods:**

This retrospective study was exempt from institutional review board approval. The American College of Surgeon National Surgical Quality Improvement Program database was used to identify patients who underwent vein stripping (VS) and endovenous procedures for treatment of chronic superficial venous disease. Endovenous options included radiofrequency ablation (RFA) and laser ablation. Data was available from 2011 to 2018 and demographic information was extracted for each patient identified by Current Procedural Terminology codes. For all racial and ethnic groups, trend lines were plotted, and the relative rate of change was determined within each specified demographic.

**Results:**

There were 21,025 patients included in the analysis. The overall mean age was 54.2 years, and the majority of patients were female (64.8%). In total, 27.9%, 55.2%, and 16.9% patients underwent VS, RFA, and laser ablation, respectively. Patients who received laser ablation were older (*P* < .001). Hispanic ethnicity was associated with significantly lower odds of receiving endovascular thermal ablation (EVTA) over VS (odds ratio [OR], 0.71; 95% confidence interval [CI], 0.64-0.78; *P* < .001). American Indian/Alaska Native patients were more likely to receive EVTA over VS (OR, 4.02; 95% CI, 2.48-6.86); similarly, Native Hawaiian/Pacific Islander patients were more likely to receive EVTA over VS, although this difference was not statistically significant (OR, 1.44; 95% CI, 0.93-2.27). On multinomial regression, Hispanic patients were less likely to receive RFA over VS, whereas American Indian/Alaskan Native patients were more likely to receive RFA over VS. In all racial and ethnic groups, the percentage of endovenous procedures increased, whereas vein stripping decreased.

**Conclusions:**

Based on a hospital-based dataset, demographic indicators, including age, sex, race, and ethnicity, are associated with differences in endovenous treatments for chronic superficial venous insufficiency suggesting disparities in obtaining minimally invasive treatment options among certain patient groups.

**Article Highlights**

• **Type of Research:** Retrospective or retrospective analysis of prospectively collected registry data from the National Surgical Quality Improvement Program
• **Key Findings:** Patients with Hispanic ethnicity have lower odds of receiving endovascular thermal ablation over venous stripping (VS). American Indian/Alaska Native patients were more likely to receive endovascular thermal ablation over VS. On multinomial regression, Hispanic patients were less likely to receive radiofrequency ablation over VS, whereas American Indian/Alaskan Native patients were more likely to receive radiofrequency ablation over VS.
• **Take Home Message:** Demographic indicators including ethnicity and race are associated with differences in endovascular treatments for chronic superficial venous disease.

In the United States, >25 million adults are affected by chronic venous disease, with an economic burden totaling >$3 billion annually, including ≤$1 billion being spent on wound care and 2 million work-days missed annually.^1^, ^2^, ^3^ Furthermore, although numerous attempts have been made at quantifying epidemiological and economic data related to superficial venous insufficiency, many real-world databases include only homogeneous patient populations, primarily involving White or Caucasian patients.^4^^,^^5^ Compression therapy is the first-line therapy for venous insufficiency symptoms, although patient compliance remains low.^6^ Venous stripping (VS), whereby typically the great saphenous vein is surgically removed, has been a long-used option with favorable long-term outcomes. However, there remains a high risk of recurrent visible veins, ongoing pain, hematoma, and saphenous neuritis.^7^^,^^8^ Over the past three decades, endovascular thermal ablation (EVTA) modalities have revolutionized superficial venous treatment, either using laser or radiofrequency ablation (RFA). With these methods, thermal energy is delivered to the vessel endothelium, resulting in disruption, fibrosis, and, eventually, ablation and vessel closure.^9^ Laser ablation has been shown to be just as effective in the long term as vein stripping, but has the additional benefits of being minimally invasive and is significantly more cost effective.^10^ RFA is another modality of thermal ablation by which occlusion of the great saphenous vein is achieved via endovenous, segmental heating. Large, randomized trials have demonstrated RFA to be associated with improved recovery rates and decreased postoperative pain scores compared with stripping, while achieving improved quality of life scores.^11^^,^^12^ In a randomized, controlled trial, it was demonstrated that RFA and laser therapy demonstrate significant improvement in Clinical, Etiology, Anatomic, Pathophysiology classification.^13^ Despite increasing interest in social determinants of health, there remains paucity in understanding disparities of access and outcomes regarding chronic venous disease.

The goal of this study was to analyze trends in treatment access for chronic superficial venous disease and to identify gender, race, and ethnicity, and age disparities in care using data from the National Surgical Quality Improvement Program (NSQIP) provided by the American College of Surgeons (ACS) in patients undergoing endovascular superficial venous treatment.

## Methods

### Patient selection

The ACS NSQIP database is the first nationally validated, risk-adjusted, outcomes-based program to measure and improve the quality of surgical care. The ACS NSQIP is designed to help hospitals to improve surgical care using risk-adjusted clinical data. The program places hospitals in the national lead in providing high-quality, effective surgical care. This study used the NSQIP to identify patients who underwent vein stripping and endovenous procedures for treatment of chronic superficial venous disease. From 2011 to 2018, demographic information was extracted for each patient identified by Current Procedural Terminology codes for chemical adhesive ablation (36482, 36483), laser ablation (36478, 36479), RFA (36475, 36476), and VS (37722, 37700, 37718, 37735).

### Statistical analyses

Patients were stratified based on age <65 years, sex, operation year, ethnicity, and race (Hispanic, White, Black, Asian, American Indian/Alaska Native, Native Hawaiian/Pacific Islander). For all racial and ethnic groups, trend lines were plotted, and the relative rate of change was determined within each specified demographic. All analyses were performed using R version 4.2.0 (R Core Team, Vienna, Austria). Data cleaning and plotting were performed using the *tidyverse* package and tables were created using *gtsummary* package. Multivariate logistic regression was used to fit a model using the demographic features shown in Table I as explanatory variables and procedure type as the dependent variable. Multinomial regression was used to simultaneously fit a model for VS vs RFA and VS vs laser ablation as the dependent variables with the same explanatory variables as used in the original logistic regression. A *P* value of <.05 was considered significant.

**Table I:** Demographics

Characteristic	VS (n = 5864)	RFA (n = 11,605)	Laser (n = 3556)	*P* value^a^
Age, years	53.3 ± 13.6	54.4 ± 13.7	55.0 ± 13.6	<.001
Age ≥65 years	1318 (22.5)	2823 (24.3)	918 (25.8)	<.001
BMI	29.9 ± 7.8	30.6 ± 8.3	30.2 ± 9.5	<.001
Sex				<.001
Female	3642 (62.1)	7686 (66.2)	2286 (64.3)	
Male	2222 (37.9)	3919 (33.8)	1270 (35.7)	
Operation year				<.001
2011	823 (14.0)	1267 (10.9)	489 (13.8)	
2012	794 (13.5)	1376 (11.9)	529 (14.9)	
2013	788 (13.4)	1395 (12.0)	502 (14.1)	
2014	688 (11.7)	1394 (12.0)	442 (12.4)	
2015	696 (11.9)	1607 (13.8)	418 (11.8)	
2016	849 (14.5)	1758 (15.1)	380 (10.7)	
2017	703 (12.0)	1518 (13.1)	444 (12.5)	
2018	523 (8.9)	1290 (11.1)	352 (9.9)	
Ethnicity				
Hispanic	940 (16.0)	1305 (11.2)	569 (16.0)	<.001
Race				
White	5107 (87.1)	9879 (85.1)	3078 (86.6)	<.001
Black	293 (5.0)	559 (4.8)	197 (5.5)	.2
Asian	228 (3.9)	527 (4.5)	116 (3.3)	.002
American Indian/Alaska Native	19 (0.3)	230 (2.0)	10 (0.3)	<.001
Native Hawaiian/Pacific Islander	30 (0.5)	105 (0.9)	23 (0.6)	.013

*BMI*, Body mass index; *EVTA*, endovascular thermal ablation; *VS*, venous stripping.

Values are mean ± standard deviation or number (%).

a Kruskal-Wallis rank sum test; Pearson's χ^2^ test.

## Results

### Patient population

**Fig 1:**
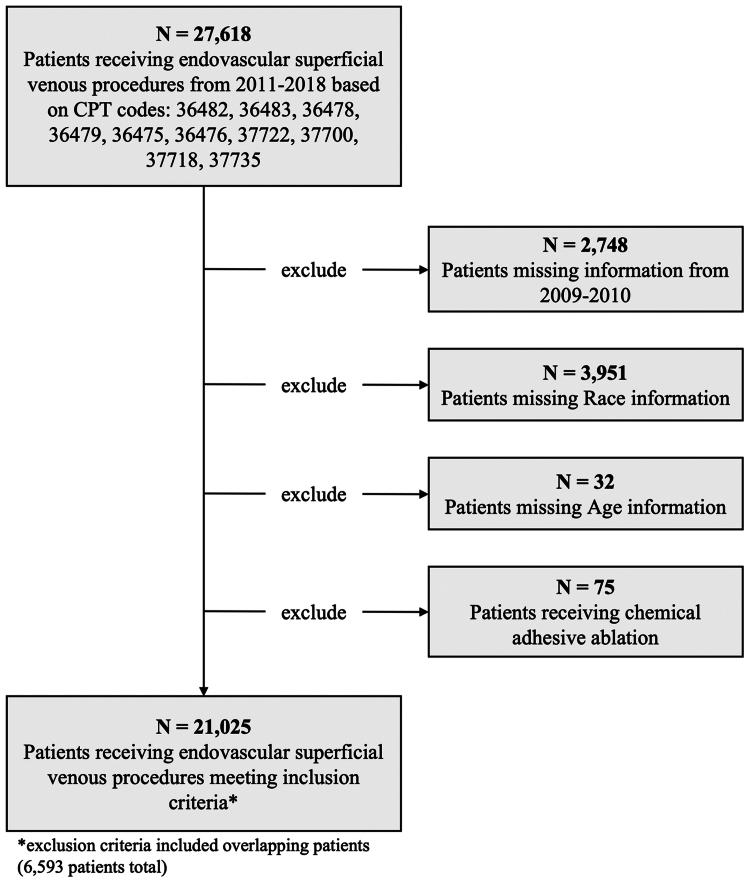
Inclusion criteria. *CPT*, Current Procedural Terminology.

There were 21,025 patients from 700 hospitals who underwent endovascular superficial venous treatment from 2011 to 2018. Current Procedural Terminology codes and exclusion criteria are demonstrated in Fig 1. The mean age was 54.2 years and 64.8% were female. Demographic breakdown is detailed in Table I. The mean age for VS, RFA, and laser ablation were 53.3, 54.5, and 55.0 years old, respectively (*P* < .001) and the percentage of patients >65 years of age were 22%, 24%, and 26%, respectively (*P* < .001). Most patients were female in all procedure cohorts.

### Association of demographic variables with venous treatment used

Logistic regression analysis (Table II) demonstrated Hispanic ethnicity was associated with significantly lower odds of receiving EVTA over VS (OR, 0.71; 95% CI, 0.64-0.78; *P* < .001). American Indian/Alaska Native patients were more likely to receive EVTA over VS (OR, 4.02; 95% CI, 2.48-6.86). Similarly, Native Hawaiian/Pacific Islander patients were more likely to receive EVTA over VS, although this result was less pronounced (OR, 1.44; 95% CI, 0.93-2.27). Neither Black nor Asian race were significantly associated with increased odds of receiving either procedure. Older age (≥65 years) was associated with increased odds of receiving EVTA (OR, 1.12; 95% CI, 1.05-1.21; *P* = .001). Male sex was associated with modestly decreased odds of receiving EVTA (OR, 0.82; 95% CI, 0.77-0.87; *P* < .001). Multinomial regression analysis (Table III) demonstrated Hispanic ethnicity was associated with significantly lower odds of receiving RFA over VS (OR, 0.63; 95% CI, 0.57-0.70; *P* < .001). American Indian/Alaska Native ancestry was associated with a>5-fold odds of receiving RFA over VS (OR, 5.05; 95% CI, 3.02-8.42). However, neither Hispanic ethnicity nor native ancestry were significantly associated with increased treatment with laser ablation over VS on multinomial regression analysis.

**Table II:** Logistic regression

Characteristic	OR (95% CI)	*P* value
Ethnicity: Hispanic	0.71 (0.64-0.78)	<.001
Race: Black	0.82 (0.65-1.05)	.11
Race: Asian	0.94 (0.74-1.21)	.6
Race: American Indian/Alaska Native	4.02 (2.48-6.86)	<.001
Race: Native Hawaiian/Pacific Islander	1.44 (0.93-2.27)	.11
Race: White	0.84 (0.69-1.01)	.070
Age ≥65 years	1.12 (1.05-1.21)	.001
Sex		
Female	—	
Male	0.82 (0.77-0.87)	<.001
BMI	1.01 (1.01-1.01)	<.001
Year operated	1.04 (1.03 -1.05)	<.001

*BMI*, Body mass index; *CI*, confidence interval; *OR*, odds ratio.

Hispanic ethnicity is associated with lower odds of receiving endovascular thermal ablation (EVTA). American Indian/Alaska Native is associated with significantly increased odds of receiving EVTA. Being male and/or <65 years old are also associated with decreased odds of receiving EVTA. Last, overall, the odds of all patients receiving EVTA increases over time.

**Table III:** Multinomial regression

Characteristic	OR^a^	*P* value
RFA		
Ethnicity		
Hispanic	0.63 (0.57-0.70)	<0.001
Race		
Black	0.80 (0.62-1.03)	0.078
Asian	1.02 (0.79-1.33)	0.9
White	0.84 (0.69-1.04)	0.11
American Indian/Alaska Native	5.04 (3.02-8.42)	<0.001
Native Hawaiian/Pacific Islander	1.57 (0.99-2.48)	0.054
Age ≥65 years	1.10 (1.02-1.18)	0.017
Sex		
Female	—	
Male	0.80 (0.75-0.85)	<0.001
BMI	1.01 (1.01-1.01)	<0.001
Year operated	1.06 (1.04-1.07)	<0.001
Laser		
Ethnicity		
Hispanic	0.96 (0.84-1.09)	0.5
Race		
Black	0.91 (0.67-1.24)	0.6
Asian	0.69 (0.49-0.98)	0.035
White	0.81 (0.63-1.05)	0.11
American Indian/Alaska Native	0.72 (0.32-1.61)	0.4
Native Hawaiian/Pacific Islander	1.04 (0.57-1.90)	0.9
Age ≥65 years	1.22 (1.10-1.34)	<0.001
Sex		
Female	—	
Male	0.90 (0.82-0.98)	0.018
BMI	1.01 (1.00-1.01)	0.035
Year operated	0.99 (0.97-1.01)	0.2

*BMI*, Body mass index; *CI*, confidence interval; *OR*, odds ratio.

Hispanic ethnicity is associated with a lower odds of receiving radiofrequency ablation (RFA) over venous stripping (VS), and Native ancestry is associated with significantly higher odds of receiving RFA over VS. Older age (≥65 years), female sex, and higher BMI are associated with significantly higher odds of endovascular thermal ablation over VS.

a Explains and summarizes the results in this table.

### Venous treatment trends over time

**Fig 2:**
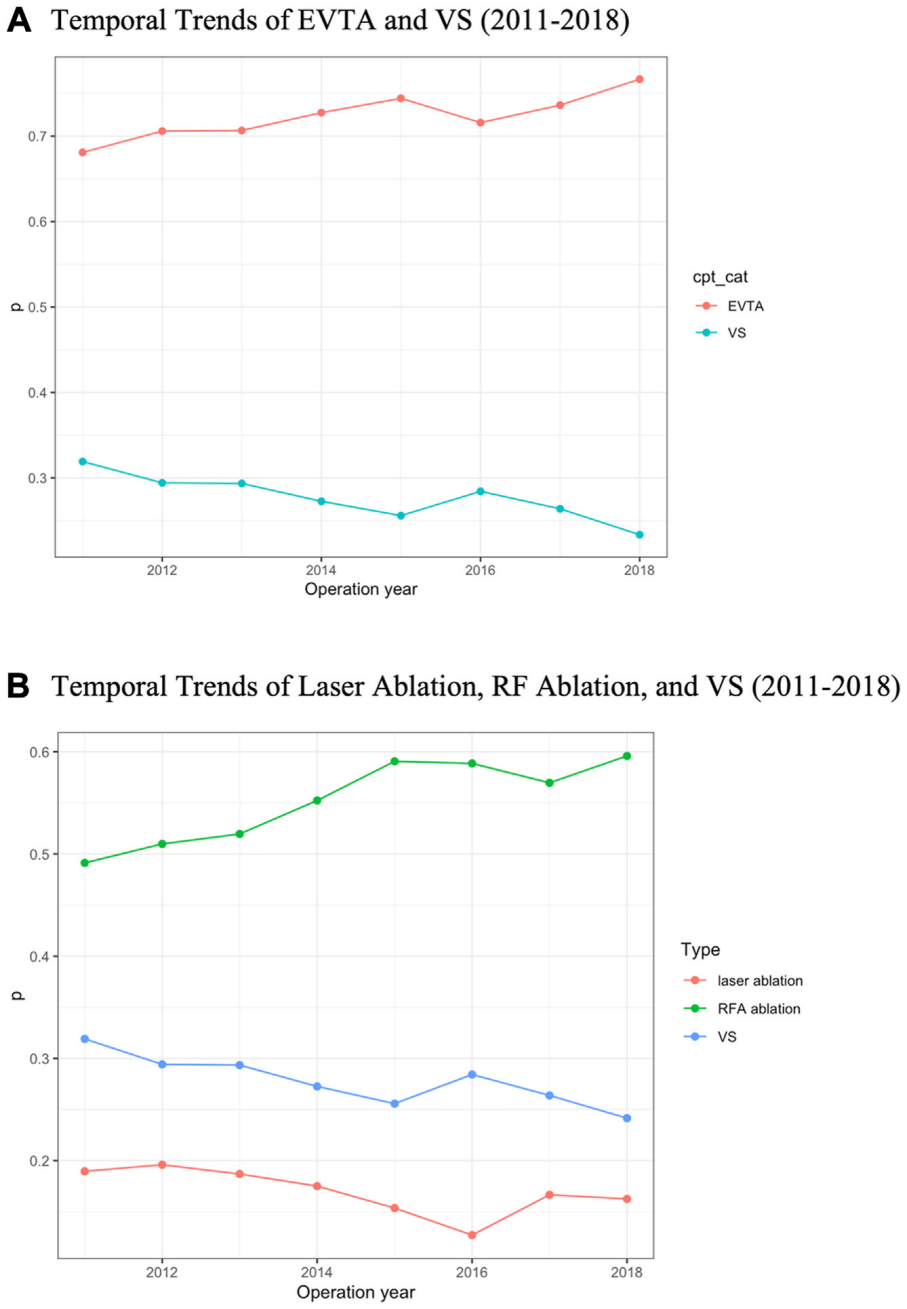
**(A)** The frequency of endovascular thermal ablation (*EVTA*) has increased from 2011 to 2018, whereas venous stripping (*VS*) has decreased. **(B)** Breakdown of EVTA into laser, radiofrequency ablation (*RFA*), and VS.

**Fig 3:**
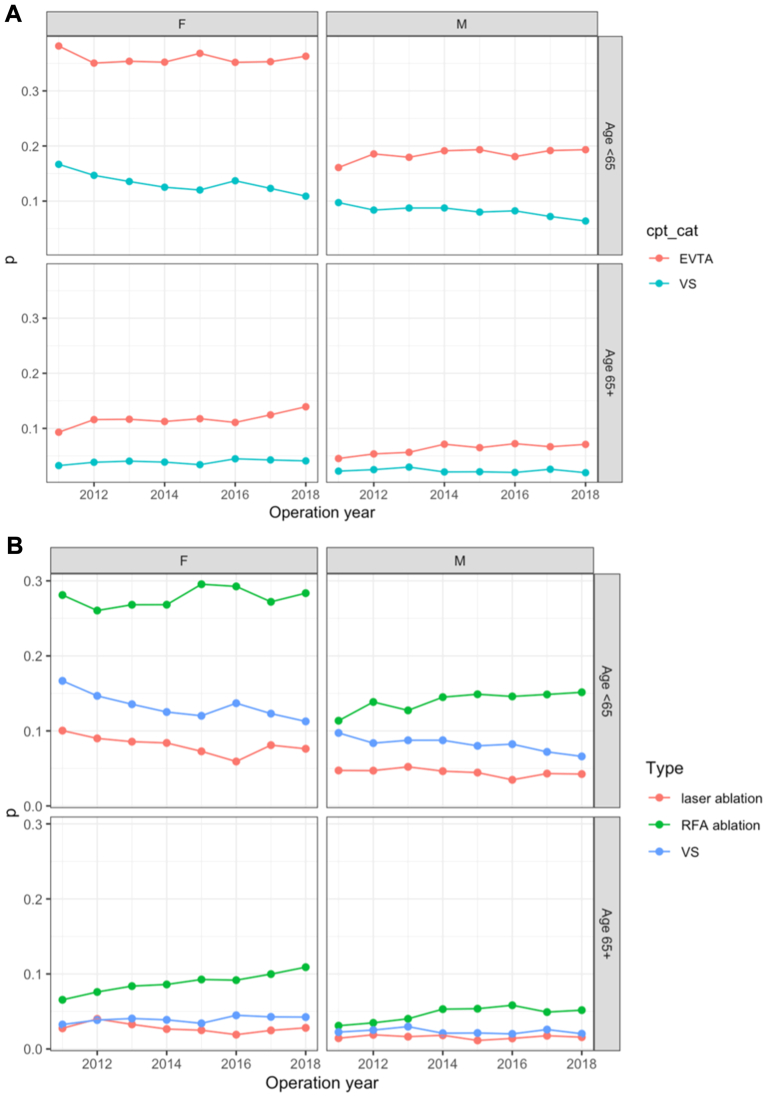
(A) Temporal trends of endovascular thermal ablation (*EVTA*) and venous stripping (*VS*) (2011-2018) by gender and age. **(B)** Temporal trends of laser ablation, radiofrequency ablation (*RFA*), and VS (2011-2018) by gender and age.

**Fig 4:**
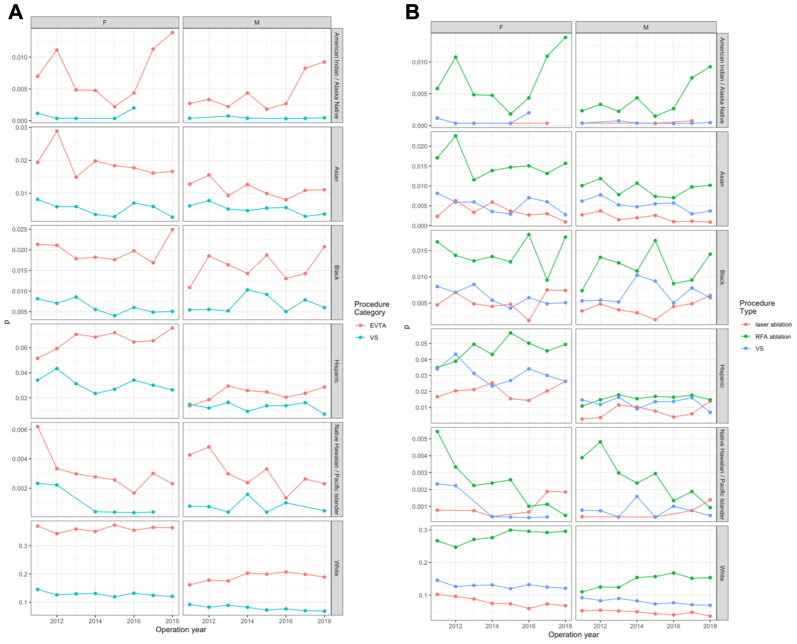
**(A)** Temporal trends of endovascular thermal ablation (*EVTA*) and venous stripping (*VS*) (2011-2018) by gender and race/ethnicity. **(B)** Temporal trends of laser ablation, radiofrequency ablation (*RFA*), and VS (2011-2018) by race/ethnicity.

Temporal analysis demonstrated frequency of EVTA increased from 2011 to 2018, whereas VS decreased (Table II, Fig 2). Regression demonstrated that, regardless of other predictors, there was a significant effect of year of treatment on receiving EVTA compared with VS. The odds of receiving EVTA increased by an average of 4% year-over-year from 2011 to 2018 (*P* < .001) (Fig 3, Fig 4). This trend was similarly seen in receiving RFA compared with VS. The odds of receiving RFA increased by an average of 6% year-over-year from 2011 to 2018 (*P* < .001), whereas the odds of receiving laser ablation remained the same over time.

## Discussion

In this national, multicenter cohort, we demonstrate that ethnicity and race are associated with differential reception of superficial venous procedures. Hispanic patients are significantly less likely to receive EVTA, specifically RFA, over VS, and Native patients are more likely to receive EVTA, specifically RFA, over VS. Additionally, we demonstrate that older age is a significant predictor of receiving EVTA. Similarly, female patients were more likely to receive EVTA. From 2011 to 2018, the use of EVTA, specifically RFA, has increased and the use of VS has decreased. This trend was observed regardless of gender, age, or race/ethnicity.^4^^,^^5^ It has been shown in other studies that RFA and EVTA are more cost effective and clinically effective compared with VS in the short and long term,^14^, ^15^, ^16^, ^17^ although VS still occurs in certain centers, likely owing to institutional practices, lack of RFA and EVTA ability, or patient preference. However, a decrease in overall VS procedures, reflected temporally in this study as well, confirm that this procedure is likely falling out of favor.^15^

Unsurprisingly, it has been shown that gender, race, ethnicity, and age have significant impacts on vascular health access and subsequent outcomes, although data regarding venous disease are lacking and most research has been conducted regarding arterial disease or fistulae outcomes.^18^, ^19^, ^20^ For example, symptom-to-door and door-to-balloon disparities in acute coronary syndrome treatment persist depending on sex; women are less likely to seek out timely care, demonstrate typical symptoms, and be diagnosed correctly with prodromal symptoms by providers; this disparity may be due to the fact that presentations differ between women and men.^21^^,^^22^ In peripheral arterial disease, Hispanic patients present initially with more severe peripheral arterial disease and are more likely to undergo major amputations.^23^^,^^24^ Black patients are also less likely to have elective endovascular aortic repair compared with Hispanic and White patients; it has been shown that Black patients are more likely to receive surgical treatment at low-volume hospitals that may not have access to advanced means of care.^25^ In dialysis access, female and Black patients are less likely to progress from a temporary access to surgically placed arteriovenous fistulae, suggesting further access barriers.^26^

It is likely that endovascular venous care follows similar trends. There may be disparities in access to care, including transportation, allowing for patients to get to centers of specialized venous care as well, where such endovascular therapies exist. For patients who must fail conservative management (eg, compression stockings) to be considered for endovascular venous therapy, it is possible that logistical barriers exist. It has further been shown that gaps in knowledge, mistrust in the medical system, discrimination from providers, and insurance barriers may play significant roles.^27^ It is likely that patients with private insurance are more able to receive advanced superficial venous care because these costs can often be out-of-pocket and such higher costs may be prohibitive despite advantages in comfort, recovery time, and long-term efficacy.^16^^,^^28^ This finding is likely augmented in the outpatient population, which this database does not capture.

Within the realm of venous disease, it has been suggested that, although racial differences in deep vein thrombosis incidence may be partly due to genetic differences between race groups (ie, sickle cell trait), it is more likely that differences are due to socioeconomic factors, including education and income.^29^^,^^30^ Black patients with pulmonary emboli who are hospitalized are more likely to be younger and present with a greater severity of disease when compared with their White counterparts. Furthermore, they are less likely to receive systemic or targeted interventions, in addition to preventative therapies.^31^

Few studies have assessed superficial venous disease although the range of venous disease (ranging from reticular veins to venous ulcers) affects about 50% of the general population, with ≤7% of men and women experiencing chronic venous insufficiency.^32^ Pappas et al^33^ assessed a large, multicenter database of patients with chronic venous disease and demonstrated that symptom presentations differ between racial groups; in their cohort, pain was more commonly the presenting symptom in African Americans, Asians, and Hispanics. Additionally, swelling symptoms were more commonly seen in African Americans and cramping symptoms were more commonly seen in Hispanics.^33^ In another large cohort from San Diego, California, the authors demonstrated non-Hispanic White patients had higher prevalence of spider veins, perhaps owing to detection bias lending to darker skin tones.^34^ Treatments and outcomes have been shown to vary between racial and ethnic groups as well. Indeed, African American patients have lower recommendation for and execution of ablation rates compared with White and Asian patients, similar to the results found in our study.^33^

To our knowledge, this study is the first to assess the association between superficial venous disease and Native ethnicity. Indeed, current guidelines outlining treatment for superficial venous disease do not acknowledge race or ethnicity and the potential structural determinants of health as a result of these factors as considerations in diagnosis or treatment.^35^^,^^36^ Most studies have focused on arterial disease considering the high rates of diabetes and other cardiovascular risk factors among American Indians and Alaska Natives.^37^, ^38^, ^39^ In the current study, we demonstrated that American Indian, Alaska Native, Native Hawaiian, and Pacific Islander patients were more likely to receive ablation over VS. Our study also demonstrated older patients were more likely to receive EVTA. We hypothesize this may be due to insurance access.^40^ Further investigation into payment structures for superficial venous disease and their effect on endovascular treatment access are warranted.

There are several limitations to this study, the greatest being that health care disparities are exceedingly complex to quantify and that the NSQIP dataset only provides 30-day data and procedural outcomes are not available in this dataset. Although this study attempts to quantify disparities in access to superficial venous disease treatments, closely related to these disparities are location, access to transportation, socioeconomic status, patient health education and health literacy, and structural racism, which are not captured in this database. As such, this study can only provide a perspective on a limited aspect of healthcare disparities within in the United States. It is further noted that many venous procedures occur in clinic settings, but the NSQIP is a hospital-based dataset and it is likely that not all venous procedures are being captured. Similarly, although the NSQIP only accounts for surgeons, other venous providers including interventional radiologists may not be fully accounted for in this dataset. However, it is plausible that, given current shortages of interventional radiologists in small and rural practices, the lack of minimally invasive venous procedures is likely among those providers as well.^41^ Additionally, it is likely that access to endovenous procedures differ depending on geographical region, and this dataset does not capture that granularity because the NSQIP does not provide that information. Last, because this is a retrospective database of surgical data, it may fail to capture patients treated at nonacademic medical centers.

## Conclusions

The management of superficial venous disease varies between racial and ethnic groups. Hispanic ethnicity is associated with significantly lower odds of receiving EVTA compared with VS, and American Indian/Alaska Native and Native Hawaiian/Pacific Islander patients are more likely to receive EVTA over VS. Other factors, including age and sex, are also associated with differences. Additionally, over time, EVTA has increased, whereas VS has decreased. Further research assessing factors contributing to disparities in access to endovascular procedures between different ethnic groups are warranted.

## Author Contributions

Conception and design: SC, AT, BD, MC, YM, SI, DD

Analysis and interpretation: SC, AT, ER, MC, DD

Data collection: SC, AT, DD

Writing the article: SC, AT, ER, YM, SI, DD

Critical revision of the article: AT, ER, BD, MC, YM, SI, DD

Final approval of the article: SC, AT, ER, BD, MC, YM, SI, DD

Statistical analysis: Not applicable

Obtained funding: Not applicable

Overall responsibility: DD

## Disclosures

None.
